# Deep brain stimulation improves electroencephalogram functional connectivity of patients with minimally conscious state

**DOI:** 10.1111/cns.14009

**Published:** 2022-11-15

**Authors:** Yuanyuan Dang, Yong Wang, Xiaoyu Xia, Yi Yang, Yang Bai, Jianning Zhang, Jianghong He

**Affiliations:** ^1^ Medical School of Chinese PLA Beijing China; ^2^ Department of Neurosurgery the First Medical Center of Chinese PLA General Hospital Beijing China; ^3^ Zhuhai UM Science and Technology Research Institute Zhuhai China; ^4^ Department of Neurosurgery the Seventh Medical Center of Chinese PLA General Hospital Beijing China; ^5^ Department of Neurosurgery, Beijing Tiantan Hospital Capital Medical University Beijing China; ^6^ Department of Basic Medical Science, School of Medicine Hangzhou Normal University Hangzhou China

**Keywords:** CRS‐R, DBS, disorders of consciousness, functional connectivity, minimally conscious state

## Abstract

**Aim:**

Deep brain stimulation (DBS) is a potential neuromodulatory therapy that enhances recovery from disorders of consciousness, especially minimally conscious state (MCS). This study measured the effects of DBS on the brain and explored the underlying mechanisms of DBS on MCS.

**Methods:**

Nine patients with MCS were recruited for this study. The neuromodulation effects of 100 Hz DBS were explored via cross‐control experiments. Coma Recovery Scale‐Revised (CRS‐R) and EEG were recorded, and corresponding functional connectivity and network parameters were calculated.

**Results:**

Our results showed that 100 Hz DBS could improve the functional connectivity of the whole, local and local–local brain regions, while no significant change in EEG functional connectivity was observed in sham DBS. The whole brain's network parameters (clustering coefficient, path length, and small world characteristic) were significantly improved. In addition, a significant increase in the CRS‐R and functional connectivity of three MCS patients who received 100 Hz DBS for 6 months were observed.

**Conclusion:**

This study showed that DBS improved EEG functional connectivity and brain networks, indicating that the long‐term use of DBS could improve the level of consciousness of MCS patients.

## Graphical abstract

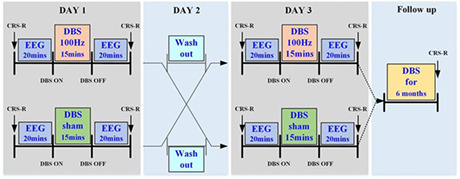

## 1 INTRODUCTION

Traumatic brain injury (TBI) is a major cause of death and disability. Although advances in critical care have significantly improved TBI‐associated mortality rates, these patients often sustain significant brain dysfunctions. Disorders of consciousness (DOC) are states where consciousness has been altered due to damages to the brain. DOC includes coma, vegetative state/unresponsive wakefulness syndrome (VS/UWS), and minimally conscious state (MCS).^1^ Studies have found that differences in the underlying pathology of DOC could predict patients' prognosis.^2^ Although VS patients may show transient periods of eye‐opening and sleep–wake cycles, their sleep patterns are significantly disorganized compared to people without DOC.^3^ The electroencephalogram (EEG) of VS patients may show slow background activity with delta rhythms and even electrocerebral inactivity, while the cortex does not respond to external stimuli.^4^ MCS patients also show sleep–wake cycles and more sleep patterns, such as spindling activity and REM sleep stage.^3^, ^5^ Compared with VS patients, MCS patients show sleep–wake cycles and more sleep patterns, such as spindling activity and REM sleep stage.^3^, ^5^ They are more responsive to stimuli, such as auditory stimuli and electrical stimulation,^6^, ^7^ show greater activation of cortical metabolic rates, and are more likely to regain consciousness with natural recovery or rehabilitation due to preservation of their brain function.^5^, ^8^ Some patients with MCS may even gradually regain consciousness and motor functions after effective rehabilitation and eventually return to society.^9^

Neuromodulatory interventions have been used in the treatment of patients with DOC.^10^, ^11^, ^12^ Transcranial direct current stimulation (tDCS), repeated transcranial magnetic stimulation (rTMS), deep brain stimulation (DBS), spinal cord electrical stimulation (SCS), and vagal nerve stimulation were shown to improve the consciousness level of these patients.^13^, ^14^, ^15^, ^16^, ^17^ DBS can directly modulate central thalamus nerve activities via electrodes in the thalamus, thus affecting the thalamus‐cortex circuit and improving the patients' consciousness.^1^ Central thalamic DBS aims at regulating the neural circuit of consciousness, and its action site is closer to the core of target of arousal regulation. Theoretically, central thalamic DBS has a higher regulation efficiency than other invasive brain stimulations.^18^ It is reported that the first DOC patient treated with DBS in 1969 showed improved EEG activity.^19^ Subsequent follow‐up studies showed the effects of DBS over several months on the recovery of consciousness.^20^, ^21^ Schiff et al.^22^ conducted a cross‐controlled experiment on an MCS patient and found that central thalamic DBS significantly promoted functional recovery from TBI and improved consciousness. Their study received great attention,^22^ inspiring many studies to use DBS to improve the consciousness of MCS patients. Kundu et al.^23^ reviewed the DBS results of DOC patients and observed that the integrity of the reticular structure, cortex and thalamic circuit influenced the effectiveness of DBS. Thus, it is necessary to accurately and objectively measure the regulation effects of DBS to further understand its underlying mechanism.

Electroencephalogram is widely used in the diagnosis of DOC and the measurement of neuromodulation effects. EEG features based on neural oscillation, nonlinear dynamics, and information theory quantify the rhythm, complexity, and coupling integration of brain activity to characterize brain functions.^24^, ^25^ Brain functional connectivity describes the synchronous activities and information interaction among different brain regions.^26^, ^27^

Theoretical works and clinical evidence suggest that consciousness depends on the capacity of brain regions to interact through functional connectivity. The information integration theory (IIT) asserts that the key explanandum of consciousness comprises of integrated information^28^ and considers that consciousness emerges from functional connectivity among distributed regions. Clinical studies have reported decreased functional connectivities in the brain during VS,^29^ anesthesia,^30^ and non‐REM sleep,^31^ especially in frontoparietal connectivity. In addition, a clear‐cut recovery of brain functional connectivity in DOC patients paralleled with the recovery of consciousness. Mutual information is a common assessment method that could address functional connectivity by calculating the interaction probability of two‐time series signals. King et al.^26^ proposed the “weighted symbolic mutual information” (wSMI), a novel measure of mutual information that measures the brain's functional connectivity during DOC. The results showed that VS patients had significantly lower brain functional connectivity than MCS patients, especially long‐distance connectivity. Li et al.^27^ proposed the permutation conditional mutual information (PCMI) and used it to measure the changes in brain information interaction during anesthesia. Liang et al.^25^ proposed genuine permutation cross mutual information (G_PCMI) to explore anesthesia mechanisms and found that the loss of consciousness caused by anesthesia was due to reduced information sharing across brain regions. The above studies not only illustrate the importance of functional connectivity in the study of consciousness but also provide evidence supporting IIT.

To study the modulation mechanism of DBS in MCS patients, we used EEG to measure cortical functional connectivity changes in MCS patients. The results provide evidence explaining the modulation mechanism of DBS and electrophysiological indicators to evaluate the modulation effects of DBS.

## 2 METHODS

### 2.1 Subjects

Patients with DOC were selected for central thalamic DBS treatment from July 2017 to July 2019 at the Seventh Medical Center of PLA General Hospital. The study inclusion criteria were (1) DOC occurred after a severe brain injury. (2) The duration of stable DOC was >3 months (for patients with traumatic injuries, the period was extended to >6 month). Stable DOC was defined as no significant improvement or deterioration of consciousness for more than 4 weeks. (3) MCS was diagnosed based on the JFK Coma Recovery Scale‐Revised (CRS‐R). Patients were excluded if they had severe brain deformation that rendered the thalamus unrecognizable, chronic neurological diseases independent from the original injury, untreated hydrocephalus, or other severe extraneurological diseases that could reduce their life expectancy to <1 year. In total, nine consecutive MCS patients were eligible for this study.

In total, nine consecutive MCS patients (five males and four females) were eligible for this study. The median age of the whole cohort was 34.3 (range, 11–49) years. The time after injury ranges from 6 to 12 months. The details regarding patient characteristics, etiology, injured brain areas, and CRS‐R scores are shown in Table 1. All patients received a thorough preoperative evaluation. An experienced clinician obtained the CRS‐R score of the patients after repeated assessments. The family members of the patients were informed of the details of the study, including the operation process, DBS postoperative care, and DBS parameter selection process, and provided signed consent for participation. The study was approved by the Ethics Committee of the Seventh Medical Center of PLA General Hospital (approval no.: No. 2017‐33).

**TABLE 1:** Patients' demographic information

Patient number	Group	Age (years)	Sex	Etiology	Injured brain sites	Post‐injury (months)	CRS‐R
1	1	25	Male	Trauma	Extensive subcortical white matter	6	12 (2‐3‐3‐1‐0‐3)^a^
2	2	35	Female	Hemorrhage	Left parietal cortex	6	9 (1‐3‐2‐1‐0‐2)
3	2	52	Male	Hemorrhage	Left basal ganglia	6	9 (1‐3‐2‐1‐0‐2)
4	1	35	Male	Hemorrhage	Brain stem	7	9 (0‐3‐3‐1‐0‐2)
5	1	45	Female	Anoxic	Extensive cortex	7	12 (2‐3‐3‐2‐0‐2)
6	2	49	Male	Trauma	Extensive subcortical white matter	12	12 (3‐1‐5‐1‐0‐2)
7	1	11	Female	Trauma	Extensive subcortical white matter	8	9 (1‐3‐2‐1‐0‐2)
8	1	26	Female	Anoxic	Extensive cortex	8	14 (2‐4‐5‐1‐0‐2)
9	2	31	Male	Trauma	Extensive subcortical white matter	6	9 (1‐3‐2‐1‐0‐2)

^a^ The score of each subscale (auditory, visual, motor, oromotor/verbal, communication, and arousal) are shown in parentheses; CRS‐R, JFK Coma Recovery Scale‐Revised.

### 2.2 Surgery

Preoperative 3D‐T1 MRI scan was performed under general anesthesia perioperatively. The bilateral centromedian‐parafascicular nuclei complexes (CM‐Pf) were targeted for all the patients. Surgery was performed under general anesthesia. Quadripolar electrode leads (L302, PINS) were implanted with the guidance of the Leksell stereotactic headframe. Intraoperative CT was used to confirm the accuracy of electrode implantation.

### 2.3 Study design

**FIGURE 1:**
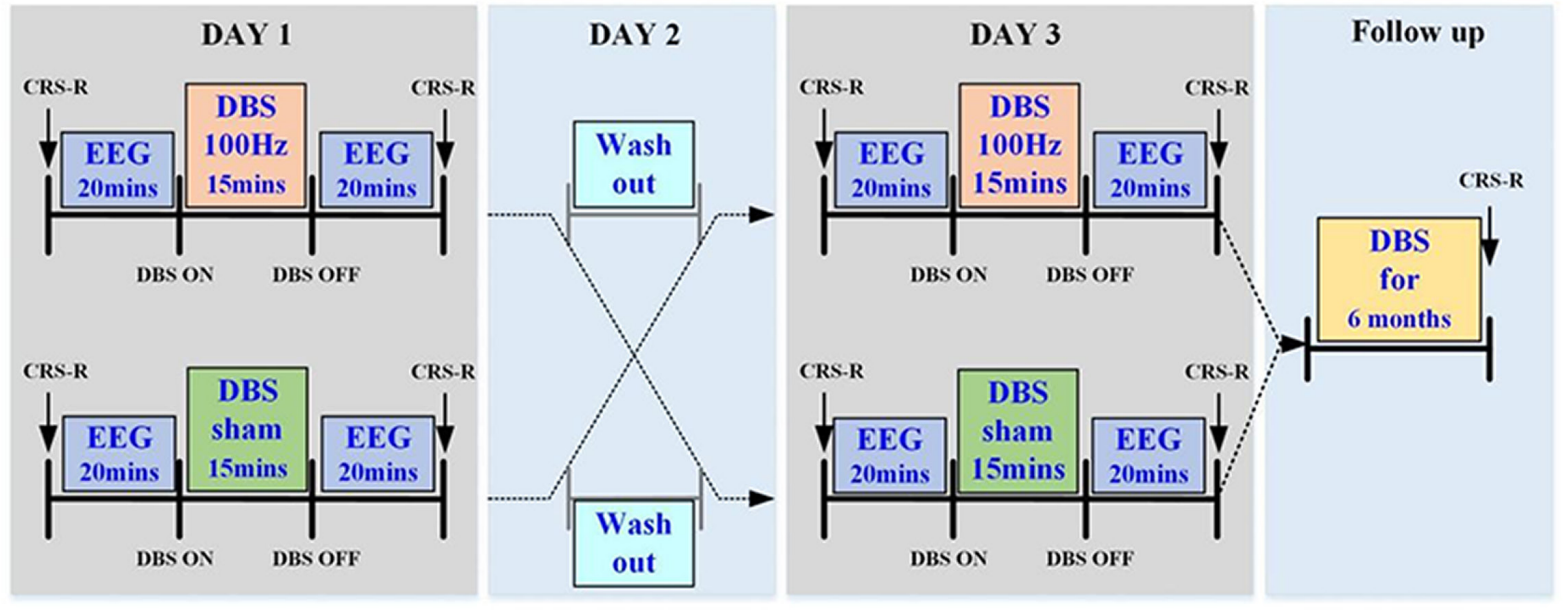
Flowchart representing the overall study design

A randomized sham‐controlled crossover study was conducted to study the short‐term effects of DBS. The nine patients were randomly divided into two groups (Table 1). In the first group, five patients received 100 Hz DBS^22^ and sham DBS on two separate days separated by 24 h. The same treatment was given to the second group but in reversed order (no. of patients = 4). The other DBS parameters included 15 min of stimulation duration, a DBS pulse width of 120 μs and a 3.0 V voltage.^22^, ^32^ CRS‐R and resting state EEG (15 min) were recorded before and immediately after DBS. The patients' follow‐up outcomes were recorded 6 months after the DBS operation using the CRS‐R scale to assess the long‐term effects of DBS. The study design is shown in Figure 1.

### 2.4 CRS‐R

CRS‐R is a stimulus–response measurement that indirectly represents the functions of corresponding brain structures and circuits through behavioral responses.^33^, ^34^ The scores range from 0 to 23 and are grouped into six subscores quantifying auditory, visual, motor, oromotor‐verbal, communication, and arousal processes. CRS‐R is widely used to assess the level of consciousness and can differentiate patients with MCS from those with VS/UWS. Patients who met the diagnostic criteria of MCS were selected for surgery. An experienced neurosurgeon measured the patient's CRS‐R through in‐hospital evaluations or home visits.

### 2.5 Neurophysiology

A 32‐lead amplifier (Brian Product, Germany) was used to record the EEG data, filtered (0.1–500 Hz) and sampled at 1000 Hz at 16‐bit resolution. The brain activity was obtained via 32 Ag/Cl electrode caps and displayed in real time through a BP viewer. The patients were required to keep their eyes open and rest, and the experiment stopped when they started sweating or felt uncomfortable. To ensure data quality, the impedance was kept below 5 KΩ.

### 2.6 EEG preprocess and analysis

EEG preprocessing was performed using EEGLAB of Matlab R2012a (Mathworks). The EEG data were verified, bad channels were removed, and data down‐sampling was performed at 250 Hz, with a band‐pass filter set at 1–45 Hz. Then, the data were segmented into epochs of 4 s. Artifactual epochs with voltages exceeding 100 μV were rejected following visual inspections. At least 45 artifact‐free epochs were retained for each data. Residual electromyographic (EMG) and electrooculographic (EOG) activity was removed by independent component analysis (ICA). Artifactual ICs were visually rejected based on time course, spectrum, and sensor topography. The EMG component showed higher frequency power, high local scalp source, and obvious spiking activity. The EOG components were symmetrical or asymmetrical, temporarily sparse, and showed intense activities in the prefrontal lobe. Lastly, the average reference data was also obtained.

### 2.7 Genuine permutation cross mutual information

The genuine permutation cross‐mutual information (G_PCMI), proposed by Liang et al.,^25^ was used to measure the functional connectivity between local brain regions. The details of the G_PCMI calculation are as follows:

1. A phase space reconstruction procedure was performed using the EEG data of two channels, *x*_*t*_ and *y*_*t*_, *t* = 1, 2,…*n*. The vectors *X*_*t*_[*x*_*t*_, *x*_*t+τ*_,…*x*_*t++mτ*_,] and *Y*_*t*_[*y*_*t*_, *y*_*t+τ*_,…*y*_*t++mτ*_,] were constructed with embedded dimension *m* and the time delay *τ*. In this study, *m* = 3, *τ* = 1.
2. *X*_*t*_ and *Y*_*t*_ were arranged in ascending order as symbols of vectors: *S*_*n*_ and *S*_*q*_, respectively. Theoretically, there were m! different types of symbolic vectors.
3. Based on the marginal probability distribution functions *p*(*x*) and *p*(*y*), the entropy of *x*_*t*_ and *y*_*t*_ were respectively defined as

(1) $$H\left(X\right)=-\sum_{j=1}^{J}p_{j}\log p_{j}$$

(2) $$H\left(Y\right)=-\sum_{j=1}^{J}p_{j}\log p_{j}$$

4 The joint entropy *H*(*X*,*Y*) was calculated based on the joint probability function, using the following equation:

(3) $$H\left(X,Y\right)=-\sum_{x\in X}\sum_{y\in Y}p\left(x,y\right)\log p\left(x,y\right)$$

5 The permutation cross‐mutual information (PCMI) of *x*_*t*_ and *y*_*t*_ was defined as

(4) $$\text{PCMI}\left(X;Y\right)=H\left(X\right)+H\left(Y\right)-H\left(X,Y\right)$$

6 The iterative amplitude‐adjusted Fourier transform method was used to generate surrogate data, $x_{t}^{\text{surr}}$ and $y_{t}^{\text{surr}}$. This study created 50 surrogate data and calculated their PCMI and ${\text{PCMI}}^{\text{surr}}$.
7 G_PCMI was calculated based on the comparison between the original PCMI (${\text{PCMI}}^{\text{original}}$) and ${\text{PCMI}}^{\text{surr}}$. The Wilcoxon signed‐rank test was used to compare ${\text{PCMI}}^{\text{original}}$ and ${\text{PCMI}}^{\text{surr}}$. If H0 = 1 and *p* < 0.001, then, G_PCMI = PCMI^original^, otherwise, G_PCMI = 0.
8 A 32 × 32 symmetric connectivity matrix of G_PCMI was obtained between 0 and 1. All G_PCMI values were sorted in ascending order, and the 70th largest value was used as the threshold. Values below this threshold were set to zero.
9 The average G_PCMI values of different brain regions, including whole brain, local brain, and across‐brain regions, were calculated. The local brain regions included the frontal lobe (FP1, FP2, FZ, F3, F4, F7, F8, FC1, FC2, FC5, and FC6), central lobe (CZ, C3, C4, T3, T4, CP1, CP2, CP5, and CP6), and parietal lobe (PZ, P3, P4, P7, P8, PO3, PO4, PO7, PO8, Oz, O1, and O2). The across‐brain regions include the frontal‐central region, central‐parietal region, and frontal–parietal region.

### 2.8 Network topological measures

We used the binarized G_PCMI matrices based on the 70th largest G_PCMI value to construct the brain network.^35^ Each node in the network corresponded to an electrode, and the G_PCMI between electrodes acted as edge weights. We calculated metrics from graph theory to characterize the brain network, including clustering coefficient, average path length, and small‐world characteristics.

The clustering coefficient indicates the degree of interconnection among nodes. The clustering coefficient of node *i* was obtained with the following equation:

(5) $${\mathrm{NCC}}_{i}=\frac{\sum_{j\neq i}\sum_{m\neq i,m\neq j}w_{\mathrm{ij}}w_{\mathrm{im}}w_{\mathrm{jm}}}{\sum_{j\neq i}\sum_{m\neq i,m\neq j}w_{\mathrm{ij}}w_{\mathrm{im}}}$$

Here, *w* represents G_PCMI. The average clustering coefficient (*C*_ave_) of the whole‐brain network was defined as

(6) $$C_{\mathrm{ave}}=\frac{1}{N}\sum_{i=1}^{N}{\mathrm{NCC}}_{i}$$

Here, *N* represents the total number of nodes in the brain network.

The average path length was another important characteristic of measure in the network. It is the average of the shortest distance (*d*_*jj*_) between all node pairs in the network. The average path length of all pairs of channels was obtained by

(7) $$L_{\mathrm{ave}}=\frac{1}{\frac{1}{N\left(N-1\right)}\sum_{i=1}^{N}\sum_{j\neq i}^{N}\left(1/d_{\mathrm{ij}}\right)}$$

A small world network is a network with a short average path length and high clustering coefficient. The small world characteristics (σ) were calculated as the ratio of average path length and clustering coefficient, based on the equation:

(8) $$\sigma =\frac{C_{\mathrm{ave}}}{L_{\mathrm{ave}}}$$

### 2.9 Statistical analysis

Statistical tests were used to compare the significance of between‐ and within‐group differences based on the G_PCMI and network metrics. The Kolmogorov–Smirnov test was performed to test the normality of the data. A paired *t*‐test was used to evaluate within‐group differences in EEG metrics before and after dual stimulation. Two‐sample *t*‐tests were performed to identify significant differences in EEG metrics between groups. Post hoc paired *t*‐test adjusted for multiple comparisons using the false discovery rate method. Statistical significance was set at *p* < 0.05.

Correlations analysis was performed by calculating the Spearman correlation coefficient for EEG features versus CRS‐R scores, with the significance level set to <0.05. EEG features included G_PMCI and Network topological measures at different time points (T0 and T1). We also calculated the Spearman correlation coefficient between CRS‐R scores and the change rate of EEG features (CR) using the following equation:

(9) $$\mathrm{CR}=\frac{\mathrm{EEG}{\text{feature}}_{T1}-\mathrm{EEG}{\text{feature}}_{T0}}{\mathrm{EEG}{\text{feature}}_{T1}+\mathrm{EEG}{\text{feature}}_{T0}}$$

## 3 RESULTS

### 3.1 CRS‐R scores

The CRS‐R of patients before and after DBS was recorded. The data showed that CRS‐R did not change after 15 min of 100 Hz DBS or sham DBS (*p* > 0.05). After 6 months of DBS, the results still showed no improvement in CRS‐R in both groups (*p* > 0.05). However, the CRS‐R scores of P4, P7, and P9 increased, with an increase from 9 (2‐1‐3‐1‐0‐2) to 12 (2‐3‐3‐2‐0‐2) for P4, 8 (1‐1‐3‐1‐0‐2) to 11 (1‐3‐4‐1‐0‐2) for P7, and 9 (1‐1‐3‐1‐0‐3) to 16 (1‐5‐5‐1‐1‐3) for P9.

### 3.2 EEG

**FIGURE 2:**
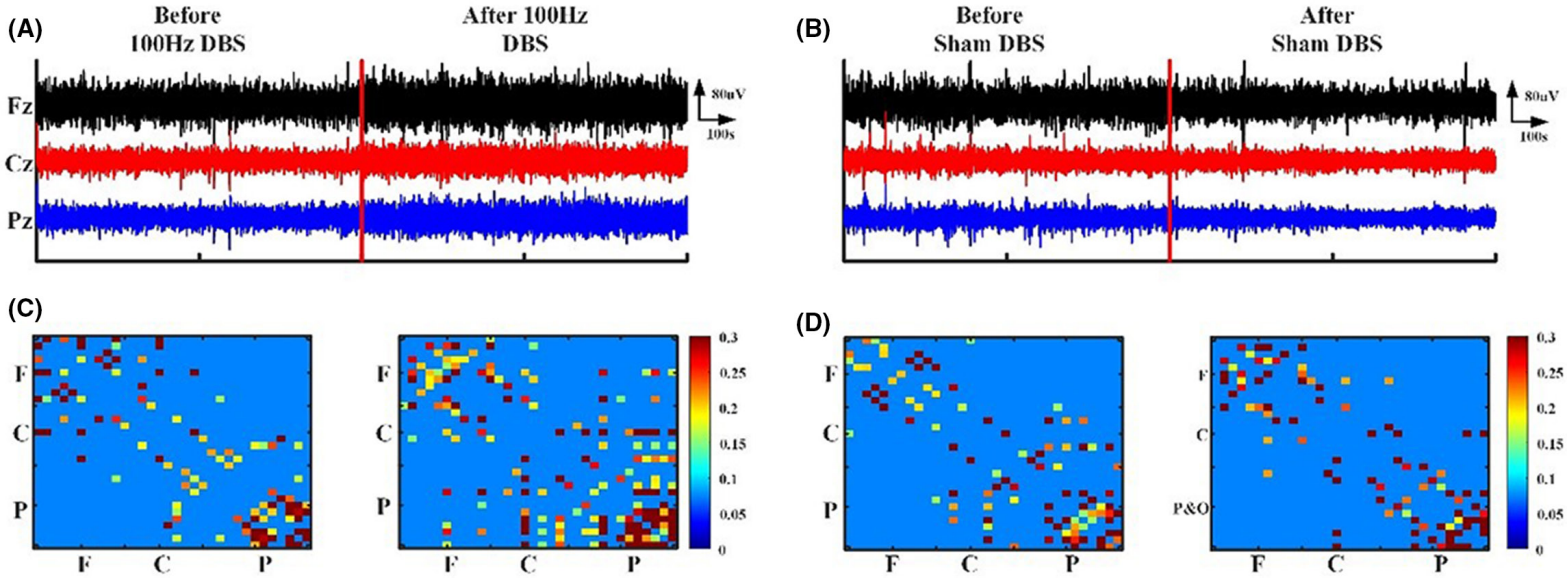
The EEG outcome for 100 Hz and sham DBS. (A, B) Raw EEG of patient P9 before and after 100 Hz and sham DBS. (C, D) The G_PCMI matrix before and after 100 Hz and sham DBS

The normal distribution of EEG metrics was evaluated using the Kolmogorov–Smirnov test (all *p* > 0.2). Figure 2 shows the EEG outcome before and after 100 Hz DBS and sham DBS for P9 (Figure 2). The raw EEG of P9 changed in amplitude. The functional connectivity of local regions (frontal and parietal lobe) and local–local regions (frontal–parietal lobe) increased for P9 patients after 100 Hz DBS (Figure 2A,C). However, almost no changes were detected with sham DBS (Figure 2B,D).

**FIGURE 3:**
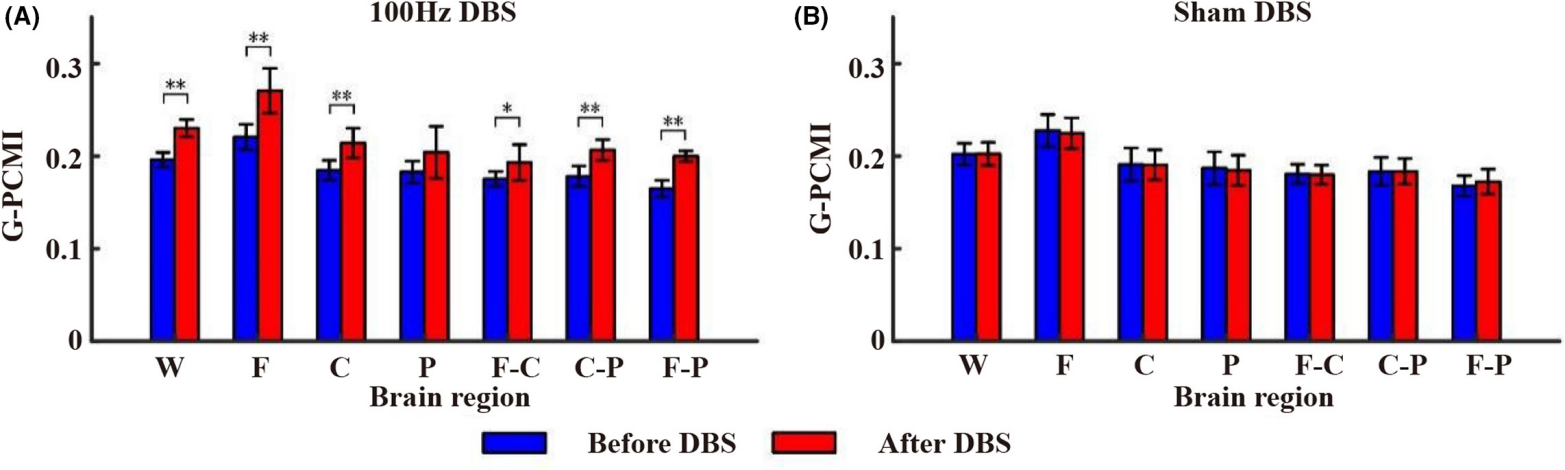
The outcome of G‐PCMI in group. Results in different brain regions (A) before and after 100 Hz DBS and (B) before and after sham DBS

The G_PCMI before and after 100 Hz DBS and sham DBS are shown in Figure 3. The results showed that the G_PCMI of the whole (*p* < 0.005), local (frontal and central lobe, *p* < 0.005) and local–local (frontal‐central lobe, *p* < 0.05; frontal–parietal lobe and central‐parietal lobe, *p* < 0.005) brain regions enhanced after 15 min of 100 Hz DBS (Figure 3A). Comparatively, 15 min of sham DBS showed no significant changes in brain functional connectivity (*p* > 0.05; Figure 3B). See Supplementary file (Data S1) for detailed data of the G_PCMI. Next, we calculated the correlation between G_PCMI and baseline CRS‐R at different time points in different brain regions. Before 100 Hz DBS, the G_PCMI of the whole brain, parietal lobe, and frontoparietal lobe was significantly correlated with baseline CRS‐R (*p* < 0.05). After 100 Hz DBS, the G_PCMI of the whole brain and the frontal‐central lobe were significantly correlated with the baseline CRS‐R (*p* < 0.05). The change rate of G_PCMI in the frontal‐central lobe and frontoparietal lobe was significantly correlated with baseline CRS‐R (*p* < 0.05; Table 2).

**TABLE 2:** Correlation results between baseline CRS‐R and EEG functional connectivity

	W	F	C	P	F‐C	C‐P	F‐P
T0	**0.67**	0.64	0.46	**0.79**	0.56	0.54	**0.83**
T1	**0.70**	0.60	0.66	0.54	**0.75**	0.48	0.47
T01	0.48	0.42	0.50	0.47	**0.76**	0.43	**0.71**

Abbreviations: T0, before DBS; T1, after DBS; T01, changes before and after DBS; W, whole brain; F, frontal lobe, C, central lobe, P, parietal lobe, F‐C, the means frontal‐central lobe; C‐P, central‐parietal lobe; F‐P means frontal–parietal lobe; CRS‐R, JFK Coma Recovery Scale‐Revised; EEG, electroencephalogram; Bold represents *p* < 0.05.

**FIGURE 4:**
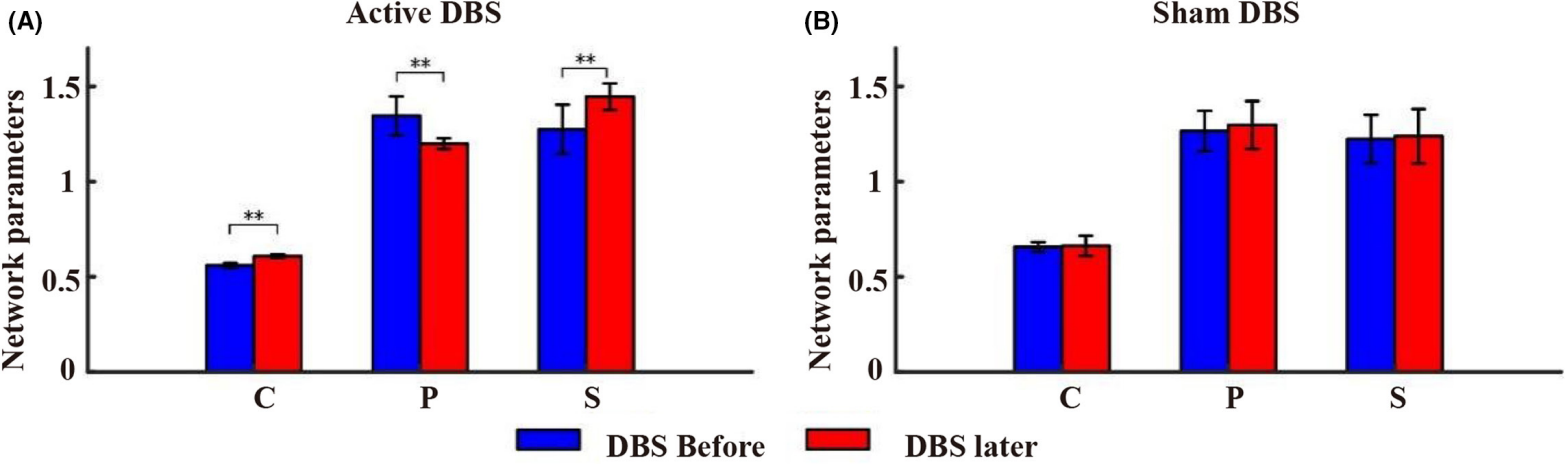
The outcome of network parameter by group. Whole brain network parameters (A) before and after 100 Hz DBS and (B) before and after sham DBS. (C) Mean clustering coefficient. L, the average path length; S, small world characteristics

Figure 4 shows the changes in network parameters of the whole brain before and after 100 Hz DBS and sham DBS (Figure 4). After 100 Hz DBS, a significant increase in clustering coefficient and small world characteristic was observed (*p* < 0.005), the average path length between network nodes decreased significantly (*p* < 0.005; Figure 4A), and there were no significant changes in network parameters from pre‐ to post‐sham stimulation (*p* > 0.05; Figure 4B). See Supplementary file (Data S1) for detailed data of the network parameters. We then calculated the correlation between network parameters and baseline CRS‐R at different time points in different brain regions. The results showed that before and after 100 Hz DBS, the three network parameters were significantly correlated with the baseline CRS‐R (*p* < 0.05). In addition, the correlation coefficients after 100 Hz DBS were higher than before 100 Hz DBS (Table 3).

**TABLE 3:** Correlation results between baseline CRS‐R and network parameters

	CC	PL	SW
T0	**0.87**	**−0.67**	**0.86**
T1	**0.96**	**−0.86**	**0.88**

Abbreviations: T0, before DBS; T1, after DBS; CC, clustering coefficient; PL, the average path length; SW, small world; Bold represents (*p* < 0.05).

**FIGURE 5:**
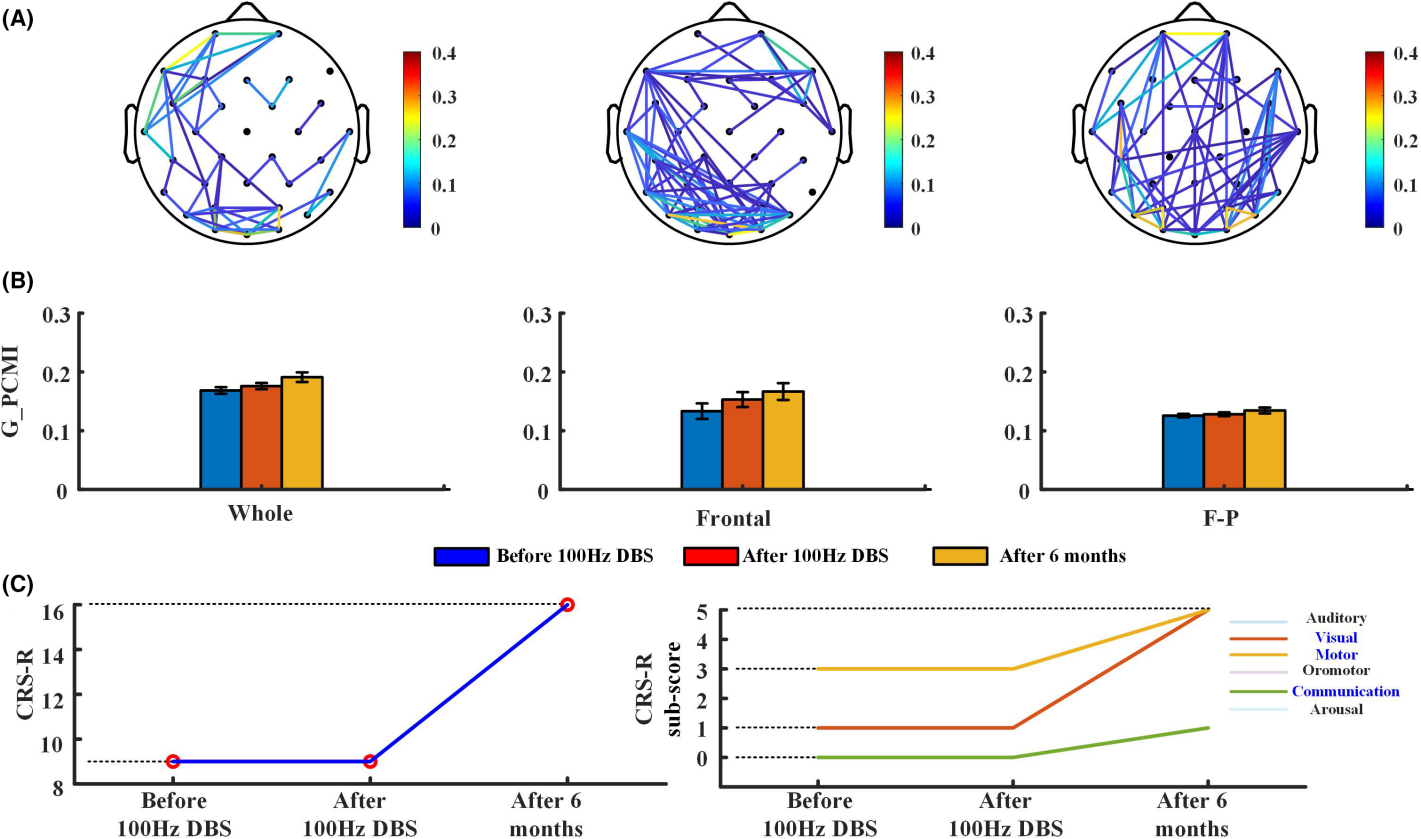
P9 outcomes. (A) Topographic map of G_PCMI at three indicated time points. (B) G_PCMI in the three regions at three indicated time points. (C) Total and sub‐score CSR‐R of G_PCMI at three indicated time points

All patients received 100 Hz DBS for 6 months. The stimulation started at 8:00 and was turned off at 20:00. After 6 months of 100 Hz DBS, the consciousness level of P4, P7, and P9 patients improved. Figures 5 and [Link], [Link] show the changes in functional connectivity and CRS‐R scores for these patients before and after 15 min DBS and after 6 months' DBS, and the results at the three time points suggest that the frontal and frontoparietal connectivity were enhanced in the three patients.

No changes were observed in the CRS‐R scores of the patients before and after DBS. After 6 months of 100 Hz DBS, the functional connectivity of P9 patients was enhanced (Figure 5A,B), and the CRS‐R increased by seven points (Figure 5C). P9 showed new behaviors in visual, motor, and communication functions. See Supplementary file (Data S1) for detailed data of the G_PCMI. P9 demonstrated the ability to recognize different objects (green ball and black pencil) and had lower accuracy in responding to autobiography questions via yes/no blinking. P9 also showed higher level behaviors related to MCS (automatic motor response). After 6 months of 100 Hz DBS in P7 patients, the whole and frontal–frontal functional connectivity improved (Figure S1A,B) and the CRS‐R increased by three points (Figure S1C). P7 showed novel behaviors in visual and motor functions, demonstrated the ability to perform smooth eye movement by following a moving green ball, and showed higher level motor behavior (object manipulation). Comparatively, the brain functional connectivity of P4 improved significantly (Figure S2A,B) and the CRS‐R increased by three points (Figure S2C). P4 also showed visual function (visual pursuit) behaviors for the first time.

## 4 DISCUSSION

In this study, G_PCMI was used to measure the changes in functional connectivity in MCS patients before and after DBS and to explore the underlying mechanism of DBS. The results show: (1) G_PCMI in whole, local, and local–local brain regions significantly improved after 15 min of 100 Hz DBS, (2) the change rate of G_PCMI wassignificantly correlated with the baseline CRS‐R, (3) the network parameters (clustering coefficient, path length and small world characteristic) of the whole brain was significantly improved, and (4) the network parameters after 100 Hz DBS demonstrated higher correlation coefficients than prior to100 Hz DBS, while no significant changes were observed after sham DBS. After 6 months of 100 Hz DBS, three patients demonstrated some behaviors related to consciousness, indicating that DBS might have long‐term effects on consciousness recovery.

Patients with DOC lose or have decreased consciousness due to severe brain injury. Previous literature indicated that exploring the mechanism of DOC could be effective in helping these patients to recover consciousness. Schiff et al.^18^ proposed the “cortical‐subcortical structure‐thalamus cortex” neural circuit to explain the mechanism of DOC, in which the thalamus was the “commander” of the consciousness circuit. Brain injury can lead to impairment in thalamus functions, resulting in a block of thalamus afferent regulation to the striatum and cortex and attenuation of the whole brain activity. Research on the mechanism of DOC provides a theoretical basis for the neuromodulation of DOC.^36^ DBS can modulate the thalamus's activity and promote consciousness recovery.^21^, ^22^ Relevant studies have confirmed that DBS can improve the activity of the thalamus, thus promoting the recovery of the brain metabolism and brain structures associated with the thalamus,^22^ such as the cortex. The cerebral cortex is an important part of the conscious neural circuit.^18^, ^37^, ^38^ Sleep and anesthesia studies have confirmed that the cortex can reflect the changes in thalamus activity with different consciousness states.^39^, ^40^ fMRI and EEG studies have shown that the cerebral cortex's functional connectivity is an important symbol of consciousness.^37^, ^38^ Tononi et al.^28^ proposed the integration theory and stated that the interaction between the thalamus and the cortex might be the basis of consciousness. Similarly, Dehaene et al.^41^ proposed the global neuronal workspace theory and believed that the cooperative work of the whole brain is key to the formation of consciousness.

This study used functional connectivity to investigate the effects of DBS on DOC. The results showed the effects of DBS on cortical connectivity, including whole, local, and local–local brain regions. A close relationship between the frontal cortex and the thalamus was previously reported.^18^, ^42^ In addition, the frontal cortex can affect other cortical activities through cortex‐cortex circuits. These results suggest that DBS could modulate brain processes through the thalamus‐cortex circuits. Moreover, the recovery of consciousness is accompanied by improved cortical activity,^42^ especially in the frontal cortex. Our findings are consistent with previous research suggesting that noninvasive neuromodulatory interventions such as tDCS and rTMS could promote consciousness recovery by targeting the frontal cortex.^13^, ^43^ Similarly, impairment in cross‐region networks might lead to loss of consciousness, especially in the frontoparietal network.^29^, ^44^ fMRI showed the correlation between frontal–parietal connectivity and consciousness level in anesthesia, sleep, and DOC.^29^, ^30^, ^31^ Laurey et al.^45^ research on DOC showed that the recovery of the frontal–parietal network represents a sign of improved consciousness. A study showed that tDCS could improve patients' consciousness by modulating the frontal–parietal connectivity. Our study confirmed the modulation effects of DBS on brain functional connectivity in patients with consciousness disorders.

Our results also showed changes in brain network characteristics from pre‐ to post‐DBS. After DBS, the clustering coefficient between the nodes of the whole brain network was enhanced, the characteristic distance of communication between nodes became smaller, and the small world characteristic of the whole brain was enhanced, indicating that the communication efficiency of the brain network was improved. Brain network is the basis of brain cognitive activities. In recent years, brain networks have been used to investigate the mechanism of consciousness. Anesthesia research showed the generation and disappearance of consciousness based on the network topology. Lee et al.^46^ found that the average path of the whole brain became longer in an anesthesia state due to propofol; that is, the disappearance of consciousness was related to the slow communication of brain network nodes. Liang et al.^25^ further confirmed that the loss of consciousness caused by propofol was due to the reduction of information exchange efficiency of brain network nodes at the cortical level. However, the brain network characteristics related to the loss of consciousness caused by brain injury are still unknown. Achard^47^ and Crone^48^ reported that the brain network characteristics in patients with consciousness disorder were lower than in normal people. Chennu et al.^35^ found a correlation between brain network characteristics and prognosis of DOC patients. This study confirmed the consciousness effects of DBS on the patients by enhancing the connection of brain network nodes, speed of information exchange, and efficiency of brain information exchange to improve consciousness activities.

DBS promoted consciousness recovery and improved visual pursuit and object manipulation in three patients. After 15 min of DBS, the functional connectivity of MCS patients enhanced, but their behavior did not improve. Schiff et al.^22^ reported that a patient could follow commands when DBS was on but failed to respond to sham DBS. It showed that 100 Hz DBS could improve the patient's consciousness, but the results in a follow‐up study were different. Considering the individual differences of patients in the two studies, the differences in the degree of brain injury might lead to different outcomes. Brain imaging studies confirmed that MCS could retain more complete brain function and thalamus‐cortical circuit, making the patients have more complex behavior. Given the relatively complete structure of MCS, such as the thalamus and frontal cortex, MCS patients have a better prognosis and are more likely to benefit from neuromodulation, such as tDCS, rTMS, and SCS. In addition, ERP and TMS‐EEG studies confirmed that MCS had more brain response activity. The results of this study show that the brain functional connectivity of MCS significantly improved after DBS. On the one hand, it confirmed that the DBS could effectively modulate MCS patients' brain activity. On the other hand, combined with the prognosis of MCS patients, it shows that the functional connectivity characterized by G_PCMI could be a marker of modulation effects in DBS.

There were some limitations to this study. Although all patients completed the CRS‐R score at 6 months follow‐up, EEG results of long‐term DBS were obtained in only three MCS patients. Despite our inclusion criteria, it was hard to disentangle spontaneous recovery from the DBS effect for long‐term follow‐up, especially for TBI patients. Future studies with a larger sample size will be needed to strengthen the power of analysis of this study.

## 5 CONCLUSION

DBS enhanced brain functional connectivity and brain network characteristics, indicating that long‐term effects of DBS could improve the consciousness level of MCS patients.

## CONFLICT OF INTEREST

All the authors declare that they have no conflict of interest.

## FUNDING INFORMATION

This research was supported by grants from the National Natural Science Foundation of China (81771128) and the Key R&D Program of Guangdong Province, China (2018B030339001).

## Supporting information


Figure S1

Figure S2

Data S1

Appendix S1

## Data Availability

The data that support the findings of this study are available on request from the corresponding author. The data are not publicly available due to privacy or ethical restrictions.

## References

[1] Naccache L . Minimally conscious state or cortically mediated state? Brain. 2018;141(4):949‐960. 29206895 10.1093/brain/awx324 PMC5888986

[2] Voss HU , Uluğ AM , Dyke JP , et al. Possible axonal regrowth in late recovery from the minimally conscious state. J Clin Invest. 2006;116(7):2005‐2011. 16823492 10.1172/JCI27021 PMC1483160

[3] Isono M , Wakabayashi Y , Fujiki MM , Kamida T , Kobayashi H . Sleep cycle in patients in a state of permanent unconsciousness. Brain Inj. 2002;16(8):705‐712. 12167195 10.1080/02699050210127303

[4] Hansotia PL . Persistent vegetative state. Review and report of electrodiagnostic studies in eight cases. Arch Neurol. 1985;42(11):1048‐1052. 4051832 10.1001/archneur.1985.04060100030015

[5] Machado C . The minimally conscious state: definition and diagnostic criteria. Neurology. 2002;59(9):1473 author reply 1473‐4, 1473; author reply 1474. 12434799

[6] Moritz CH , Black SL . fMRI reveals large‐scale network activation in minimally conscious patients. Neurology. 2005;65(11):1843. 10.1212/01.wnl.0000200030.64810.53 16344544

[7] Bai Y , Xia X , Kang J , Yang Y , He J , Li X . TDCS modulates cortical excitability in patients with disorders of consciousness. Neuroimage Clin. 2017;15:702‐709. 28702347 10.1016/j.nicl.2017.01.025 PMC5487253

[8] Laureys S , Faymonville M , Ferring M . Differences in brain metabolism between patients in coma, vegetative state, minimally conscious state and locked‐in syndrome. Neuroimage. 2003;13:806.

[9] Dang Y , Ping J , Guo Y , et al. Cranioplasty for patients with disorders of consciousness. Ann Palliat Med. 2021;10(8):8889‐8899. 34488376 10.21037/apm-21-1822

[10] Xiang X‐J , Sun L‐Z , Xu C‐B , et al. The clinical effect of vagus nerve stimulation in the treatment of patients with a minimally conscious state. J Neurorestoratol. 2020;8(3):160‐171.

[11] Yang Y , Xu L , Xie R , et al. A meta‐analysis on the efficiency of the time window of hyperbaric oxygen treatment on disorders of consciousness in China. J Neurorestoratol. 2020;8(4):270‐280.

[12] Li Y , He J , Yang B , et al. Clinical diagnosis guidelines and neurorestorative treatment for chronic disorders of consciousness (2021 China version). J Neurorestoratol. 2021;9(1):50‐59.

[13] Thibaut A , Bruno MA , Ledoux D , Demertzi A , Laureys S . tDCS in patients with disorders of consciousness: sham‐controlled randomized double‐blind study. Neurology. 2014;82(13):1112‐1118. 24574549 10.1212/WNL.0000000000000260

[14] Yamamoto T , Katayama Y , Obuchi T , Kobayashi K , Oshima H , Fukaya C . Spinal cord stimulation for treatment of patients in the minimally conscious state. Neurol Med Chir. 2012;52(7):475‐481. 10.2176/nmc.52.475 22850495

[15] Schiff ND . Central thalamic contributions to arousal regulation and neurological disorders of consciousness. Ann N Y Acad Sci. 2008;1129:105‐118. 18591473 10.1196/annals.1417.029

[16] Pape TL , Rosenow JM , Patil V , et al. RTMS safety for two subjects with disordered consciousness after traumatic brain injury. Brain Stimul. 2014;7(4):620‐622. 24836500 10.1016/j.brs.2014.03.007

[17] Yu YT , Yang Y , Wang LB , et al. Transcutaneous auricular vagus nerve stimulation in disorders of consciousness monitored by fMRI: the first case report. Brain Stimul. 2017;10(2):328‐330. 28017322 10.1016/j.brs.2016.12.004

[18] Schiff ND . Recovery of consciousness after brain injury: a mesocircuit hypothesis. Trends Neurosci. 2010;33(1):1‐9. 19954851 10.1016/j.tins.2009.11.002 PMC2931585

[19] Hassler R , Ore GD , Bricolo A , Dieckmann G , Dolce G . EEG and clinical arousal induced by bilateral long‐term stimulation of pallidal systems in traumatic vigil coma. Electroencephalogr Clin Neurophysiol. 1969;27(7):689‐690. 10.1016/0013-4694(69)91313-3 4187363

[20] Tsubokawa T , Yamamoto T , Katayama Y , Hirayama T , Maejima S , Moriya T . Deep‐brain stimulation in a persistent vegetative state: follow‐up results and criteria for selection of candidates. Brain Inj. 1990;4(4):315‐327. 2252964 10.3109/02699059009026185

[21] Yamamoto T , Katayama Y , Kobayashi K , Oshima H , Fukaya C , Tsubokawa T . Deep brain stimulation for the treatment of vegetative state. Eur J Neurosci. 2010;32(7):1145‐1151. 21039954 10.1111/j.1460-9568.2010.07412.x

[22] Schiff ND , Giacino JT , Kalmar K , et al. Behavioural improvements with thalamic stimulation after severe traumatic brain injury. Nature. 2007;448(7153):600‐603. 17671503 10.1038/nature06041

[23] Kundu B , Brock AA , Englot DJ , Butson CR , Rolston JD . Deep brain stimulation for the treatment of disorders of consciousness and cognition in traumatic brain injury patients: a review. Neurosurg Focus. 2018;45(2):E14. 10.3171/2018.5.FOCUS18168 PMC6193266 30064315

[24] Engemann DA , Raimondo F , King JR , et al. Robust EEG‐based cross‐site and cross‐protocol classification of states of consciousness. Brain. 2018;141(11):3179‐3192. 30285102 10.1093/brain/awy251

[25] Liang Z , Cheng L , Shao S , et al. Information integration and mesoscopic cortical connectivity during propofol anesthesia. Anesthesiology. 2020;132(3):504‐524. 31714269 10.1097/ALN.0000000000003015

[26] King JR , Sitt JD , Faugeras F , et al. Information sharing in the brain indexes consciousness in noncommunicative patients. Curr Biol. 2013;23(19):1914‐1919. 24076243 10.1016/j.cub.2013.07.075 PMC5635964

[27] Li X , Ouyang G . Estimating coupling direction between neuronal populations with permutation conditional mutual information. Neuroimage. 2010;52(2):497‐507. 20452438 10.1016/j.neuroimage.2010.05.003

[28] Tononi G . An information integration theory of consciousness. BMC Neurosci. 2004;5:42. 15522121 10.1186/1471-2202-5-42 PMC543470

[29] Boly M , Tshibanda L , Vanhaudenhuyse A , et al. Functional connectivity in the default network during resting state is preserved in a vegetative but not in a brain dead patient. Hum Brain Mapp. 2009;30(8):2393‐2400. 19350563 10.1002/hbm.20672 PMC6870763

[30] Boveroux P , Vanhaudenhuyse A , Bruno MA , et al. Breakdown of within‐ and between‐network resting state functional magnetic resonance imaging connectivity during propofol‐induced loss of consciousness. Anesthesiology. 2010;113(5):1038‐1053. 20885292 10.1097/ALN.0b013e3181f697f5

[31] Sämann PG , Wehrle R , Hoehn D , et al. Development of the brain's default mode network from wakefulness to slow wave sleep. Cereb Cortex. 2011;21(9):2082‐2093. 21330468 10.1093/cercor/bhq295

[32] Magrassi L , Maggioni G , Pistarini C , et al. Results of a prospective study (CATS) on the effects of thalamic stimulation in minimally conscious and vegetative state patients. J Neurosurg. 2016;125(4):972‐981. 26745476 10.3171/2015.7.JNS15700

[33] Giacino JT , Kalmar K , Whyte J . The JFK Coma Recovery Scale‐Revised: measurement characteristics and diagnostic utility. Arch Phys Med Rehabil. 2004;85(12):2020‐2029. 15605342 10.1016/j.apmr.2004.02.033

[34] Thier P , Ilg UJ . The neural basis of smooth‐pursuit eye movements. Curr Opin Neurobiol. 2005;15(6):645‐652. 16271460 10.1016/j.conb.2005.10.013

[35] Chennu S , Annen J , Wannez S , et al. Brain networks predict metabolism, diagnosis and prognosis at the bedside in disorders of consciousness. Brain. 2017;140(8):2120‐2132. 28666351 10.1093/brain/awx163

[36] Thibaut A , Schiff N , Giacino J , Laureys S , Gosseries O . Therapeutic interventions in patients with prolonged disorders of consciousness. Lancet Neurol. 2019;18(6):600‐614. 31003899 10.1016/S1474-4422(19)30031-6

[37] Rosanova M , Gosseries O , Casarotto S , et al. Recovery of cortical effective connectivity and recovery of consciousness in vegetative patients. Brain. 2012;135(Pt 4):1308‐1320. 22226806 10.1093/brain/awr340 PMC3326248

[38] Laureys S . Functional neuroimaging in the vegetative state. NeuroRehabilitation. 2004;19(4):335‐341. 15671588

[39] Massimini M , Ferrarelli F , Huber R , Esser SK , Singh H , Tononi G . Breakdown of cortical effective connectivity during sleep. Science. 2005;309(5744):2228‐2232. 16195466 10.1126/science.1117256

[40] Ferrarelli F , Massimini M , Sarasso S , et al. Breakdown in cortical effective connectivity during midazolam‐induced loss of consciousness. Proc Natl Acad Sci USA. 2010;107(6):2681‐2686. 20133802 10.1073/pnas.0913008107 PMC2823915

[41] Dehaene S , Changeux JP . Experimental and theoretical approaches to conscious processing. Neuron. 2011;70(2):200‐227. 21521609 10.1016/j.neuron.2011.03.018

[42] Van der Werf YD , Witter MP , Groenewegen HJ . The intralaminar and midline nuclei of the thalamus. Anatomical and functional evidence for participation in processes of arousal and awareness. Brain Res Brain Res Rev. 2002;39(2–3):107‐140. 12423763 10.1016/s0165-0173(02)00181-9

[43] Xia X , Bai Y , Zhou Y , et al. Effects of 10 Hz repetitive transcranial magnetic stimulation of the left dorsolateral prefrontal cortex in disorders of consciousness. Front Neurol. 2017;8:182. 28515709 10.3389/fneur.2017.00182 PMC5413493

[44] Fernández‐Espejo D , Junque C , Cruse D , et al. Combination of diffusion tensor and functional magnetic resonance imaging during recovery from the vegetative state. BMC Neurol. 2010;10:77. 20815871 10.1186/1471-2377-10-77 PMC2941677

[45] Laureys S , Faymonville ME , Luxen A , Lamy M , Franck G , Maquet P . Restoration of thalamocortical connectivity after recovery from persistent vegetative state. Lancet. 2000;355(9217):1790‐1791. 10832834 10.1016/s0140-6736(00)02271-6

[46] Lee H , Mashour GA , Noh GJ , Kim S , Lee U . Reconfiguration of network hub structure after propofol‐induced unconsciousness. Anesthesiology. 2013;119(6):1347‐1359. 24013572 10.1097/ALN.0b013e3182a8ec8c PMC3873632

[47] Achard S , Delon‐Martin C , Vértes PE , et al. Hubs of brain functional networks are radically reorganized in comatose patients. Proc Natl Acad Sci USA. 2012;109(50):20608‐20613. 23185007 10.1073/pnas.1208933109 PMC3528500

[48] Crone JS , Soddu A , Höller Y , et al. Altered network properties of the fronto‐parietal network and the thalamus in impaired consciousness. Neuroimage Clin. 2014;4:240‐248. 24455474 10.1016/j.nicl.2013.12.005 PMC3895618

