# Supplementary figures and images for: Deep brain stimulation improves electroencephalogram functional connectivity of patients with minimally conscious state
Source: CNS Neurosci Ther. 2022 Nov 15;29(1):344–53. doi: 10.1111/cns.14009 (PMC9804046; doi:10.1111/cns.14009)

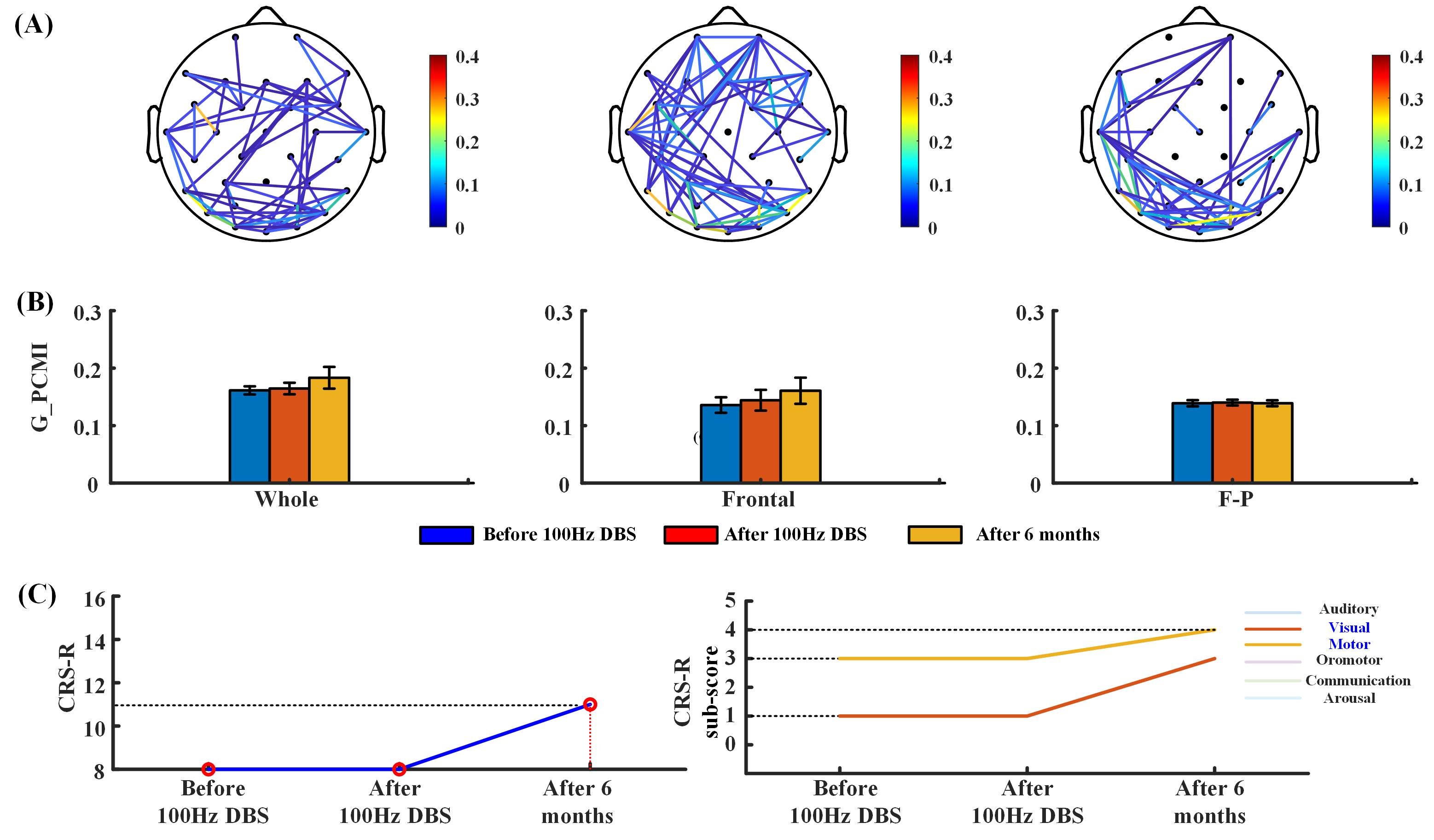

Supplement: Supplementary file 1 — Figure S1 [file CNS-29-344-s004.tif]

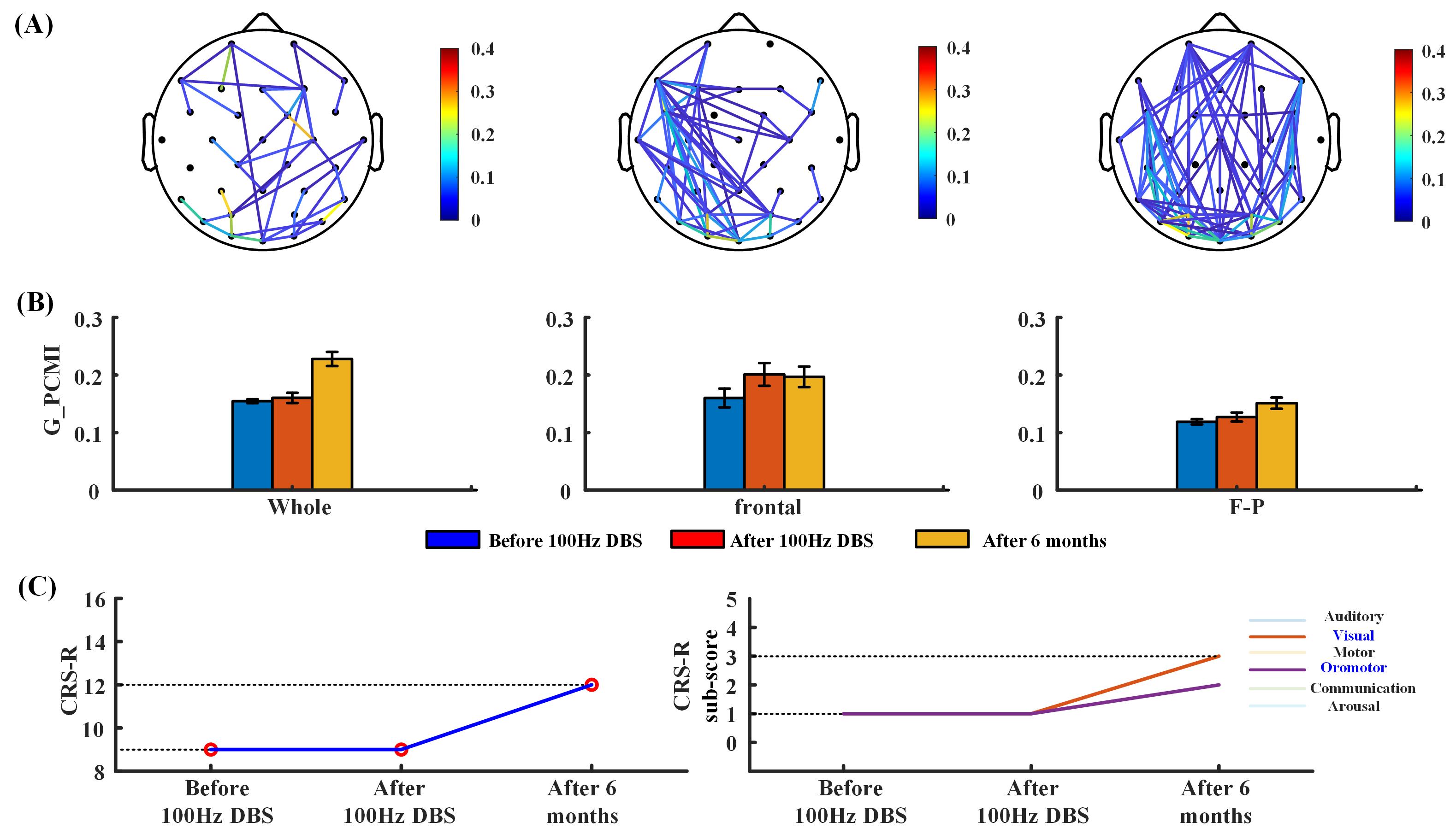

Supplement: Supplementary file 2 — Figure S2 [file CNS-29-344-s002.tif]
