# Supplementary material for: Deep brain stimulation improves electroencephalogram functional connectivity of patients with minimally conscious state
Source: CNS Neurosci Ther. 2022 Nov 15;29(1):344–53. doi: 10.1111/cns.14009 (PMC9804046; doi:10.1111/cns.14009)
Supplement: Supplementary file 4 — Appendix S1 [file CNS-29-344-s001.docx]

Figure legends

Figure S1 P7 outcomes. (A) Topographic map of G_PCMI at three indicated time points. (B) G_PCMI in three regions at three indicated time points. (C) Total and subscore CSR-R of G_PCMI at three indicated time points.

Figure S2 P4 outcomes. (A) Topographic map of G_PCMI at three indicated time points. (B) G_PCMI in three regions at three indicated time points. (C) Total and subscore CSR-R of G_PCMI at three indicated time points.
